# Antiproliferative and proapoptotic activity of GUT-70 mediated through potent inhibition of Hsp90 in mantle cell lymphoma

**DOI:** 10.1038/sj.bjc.6606007

**Published:** 2010-12-07

**Authors:** L Jin, Y Tabe, S Kimura, Y Zhou, J Kuroda, H Asou, T Inaba, M Konopleva, M Andreeff, T Miida

**Affiliations:** 1 Department of Clinical Laboratory Medicine, Juntendo University School of Medicine, 2-1-1 Hongo, Bunkyo-ku, Tokyo 113-8421, Japan; 2 Sportology Center, Juntendo University School of Medicine, 2-1-1 Hongo, Bunkyo-ku, Tokyo 113-8421, Japan; 3 Division of Hematology, Respiratory Medicine and Oncology, Department of Internal Medicine, Faculty of Medicine, Saga University, 5-1-1 Nabeshima, Saga 849-8501, Japan; 4 Division of Hematology and Oncology, Department of Medicine, Kyoto Prefectural University of Medicine, 465 Kajii-cho, Kamigyo-ku, Kyoto 602-8566, Japan; 5 Department of Molecular Oncology and Leukemia Program Project, Research Institute for Radiation Biology & Medicine, Hiroshima University, 1-2-3 Kasumi, Minami-ku, Hiroshima 734-8553, Japan; 6 Department of Leukemia, The University of Texas MD Anderson Cancer Center, 1515 Holcombe Blvd., Houston, TX 77030, USA; 7 Department of Stem Cell Transplantation and Cellular Therapy, The University of Texas MD Anderson Cancer Center, 1515 Holcombe Blvd., Houston, TX 77030, USA

**Keywords:** GUT-70, mantle cell lymphoma, apoptosis, p53, Hsp90, coumarin

## Abstract

**Background:**

Mantle cell lymphoma (MCL) is an aggressive B-cell lymphoma with poor prognosis, requiring novel anticancer strategies.

**Methods:**

Mantle cell lymphoma cell lines with known *p53* status were treated with GUT-70, a tricyclic coumarin derived from *Calophyllum brasiliense*, and the biological and biochemical consequences of GUT-70 were studied.

**Results:**

GUT-70 markedly reduced cell proliferation/viability through G_1_ cell cycle arrest and increased apoptosis, with greater sensitivity in mutant (mt)-*p53-*expressing MCL cells than in wild-type (wt)-*p53-*bearing cells. Mechanistically, GUT-70 showed binding affinity to heat-shock protein 90 (Hsp90) and ubiquitin-dependent proteasomal degradation of Hsp90 client proteins, including cyclin D1, Raf-1, Akt, and mt-p53. Depletion of constitutively overexpressed cyclin D1 by GUT-70 was accompanied by p27 accumulation and decreased Rb phosphorylation. GUT-70 induced mitochondrial apoptosis with Noxa upregulation and Mcl-1 downregulation in mt-*p53* cells, but Mcl-1 accumulation in wt-*p53* cells. Noxa and Mcl-1 were coimmunoprecipitated, and activated BAK. Treatment with a combination of GUT-70 and bortezomib or doxorubicin had synergistic antiproliferative effects in MCL cells that were independent of *p53* status.

**Conclusion:**

GUT-70 has pronounced antiproliferative effects in MCL with mt-*p53*, a known negative prognostic factor for MCL, through Hsp90 inhibition. These findings suggest that GUT-70 has potential utility for the treatment of MCL.

Mantle cell lymphoma (MCL) is characterised by an aggressive clinical course, with rapid relapse after an initial response or primary resistance to standard chemotherapy (Jares *et al*, 2007). The t(11,14)(q13;32) translocation of MCL leads to overexpression of cyclin D1, which is believed to be associated with oncogenesis by causing instability of the G_1_/S checkpoint through promotion of cyclin-dependent kinase activity and through sequestration of the Cip/Kip family of cyclin-dependent kinase inhibitors (Sherr and Roberts, 1999; Quintanilla-Martinez *et al*, 2003). These activities facilitate phosphorylation and inactivation of the retinoblastoma (Rb) G_1_/S checkpoint protein, resulting in cell cycle progression. It has been demonstrated, however, that overexpression of cyclin D1 itself is not sufficient for development of MCL, suggesting that additional genetic events might be necessary for oncogenesis (Bodrug *et al*, 1994), particularly as apoptosis-related genes such as *p53*, *INK4a/ARF*, and *ATM* are dysregulated in MCL (Fernàndez *et al*, 2005; Greiner *et al*, 2006).

In MCL, mutation/overexpression of *p53* is reported as an adverse prognostic indicator (Jares *et al*, 2007). As many of the antitumour effects mediated by chemotherapeutic agents depend on a *p53*-related pathway, resistance to chemotherapy often develops through impaired *p53* signalling (Döhner *et al*, 1995). The 26S proteasome inhibitor bortezomib retains activity in *p53*-mutant (mt-*p53*) cells and has demonstrated single-agent efficacy in relapsed or refractory MCL, which is, however, based mainly on prolonged response rather than on an increase in ultimate survival rate (Goy *et al*, 2009).

Therefore, development of novel compounds that target *p53*-independent signalling pathways is of considerable interest in the treatment of this disease.

We have reported that the newly discovered anticancer agent GUT-70, a natural product derived from the stem bark of *Calophyllum brasiliense*, demonstrated cytotoxic efficacy in human leukaemic cells (Kimura *et al*, 2005). GUT-70 (Figure 1), characterised as a tricyclic coumarin with the formula 5-methoxy-2,2-dimethyl-6-(2-methyl-1-oxo-2-butenyl) -10-propyl-2*H*,8*H*-benzo[1,2-*b*;3,4-*b*′]dipyran-8-one (C_23_H_26_O_5_), significantly inhibited leukaemic cell growth with a median inhibitory concentration (IC_50_) of 2-5 *μ*M without repressing colony formation by normal haematopoietic progenitors or proliferation of normal human hepatocytes at concentrations up to 30 *μ*M (Kimura *et al*, 2005).

Coumarin antibiotics have been reported to bind the newly discovered C-terminal ATP binding site of 90 kDa heat-shock protein (Hsp90), a molecular chaperone responsible for the folding and conformational maintenance of client proteins (Marcu *et al*, 2000; Issacs *et al*, 2003; Pratt and Toft, 2003; Donnelly *et al*, 2008). Hsp90 inhibition results in degradation of misfolded Hsp90 clients through ubiquitination, followed by proteasome-mediated hydrolysis (Zhang *et al*, 2004). As many of the Hsp90 client proteins contribute to cancer cell proliferation, Hsp90 has emerged as a promising target for cancer chemotherapy (Issacs *et al*, 2003; Donnelly *et al*, 2008).

In this study, GUT-70 demonstrated antiproliferative and proapoptotic activities with more prominent efficacy in mt-*p53-*bearing MCL cells than in those with wild-type (wt) *p53*. GUT-70 showed binding affinity to Hsp90, and reduced expression of Hsp90 client proteins such as mt-p53, Raf-1, cyclin D1, and Akt. The intrinsic apoptotic pathway was activated by GUT-70 through upregulation of Noxa and BAK activation. The combination of GUT-70 with bortezomib or doxorubicin yielded synergistic antiproliferative effects independent of *p53* status. These findings indicate possible efficacy and a rationale for further exploration of GUT-70 as a new therapeutic strategy for MCL.

## Materials and methods

### Cell lines and culture conditions

Four MCL cell lines were used in this study: JVM-2 (Melo *et al*, 1986), Granta 519 (Jadayel *et al*, 1997) and MINO (Lai *et al*, 2002) were kindly provided by Dr M Raffeld, and Jeko-1 (Raynaud *et al*, 1993) was a gift from Dr M Seto. Granta 519 and JVM-2 express wt-*p53*, whereas Jeko-1 and MINO express *p53* mutations (Jeko-1, loss of *p53* expression; MINO, mutation at codon 147 (valine → glycine)) (Raynaud *et al*, 1993; Lai *et al*, 2002). JVM-2, Jeko-1 and MINO were cultured in RPMI 1640 medium containing 15% fetal bovine serum (FBS) and 1% penicillin/streptomycin. Granta 519 was grown in Dulbecco's modified Eagle's medium (DMEM) supplemented with 15% FBS. Cells were first acclimated in RPMI 1640 or DMEM containing 5% FBS for 16 h before exposure to GUT-70 (Nippon Shinyaku, Kyoto, Japan) (Kimura *et al*, 2005). Control cells were treated with an equivalent amount of dimethyl sulphoxide (DMSO). Doxorubicin was obtained from Sigma (St Louis, MO, USA) and bortezomib was provided by Millennium (Cambridge, MA, USA). Human osteosarcoma cell line U2OS transfected with the histone cluster 1 (*H2BK)* and enhanced green fluorescent protein (*EGFP*) genes, U2OS-H2BK-EGFP, was grown in DMEM supplemented with 10% FBS and used for morphological observation. U2OS expresses wt-*p53* (Flørenes *et al*, 1994).

### Cell viability/proliferation assay

Cell viability was assessed by the Trypan blue dye exclusion method, and cell proliferation was determined by the CellTiter 96 AQueous One Solution Cell Proliferation Assay (MTS; Promega, Madison, WI, USA).

### Apoptosis analysis

Apoptotic cell death was evaluated through annexin V (Roche Diagnostic, Indianapolis, IN, USA) and propidium iodide (PI) positivities by a FACScan flow cytometer and Cell Quest software (Becton Dickinson Immunocytometry Systems, San Jose, CA, USA). The extent of drug-specific apoptosis was assessed by the following formula: % specific apoptosis=(test−control) × 100/(100−control).

### Flow cytometric analysis of cell cycle and BAK activation

Cell cycle distribution was determined by flow cytometric analysis of PI-stained nuclei. DNA content was determined by FACScan flow cytometer and CellQuest software. BAK activation was analyzed as previously described (Samraj *et al*, 2006). Briefly, cells were fixed and permeabilized using the DAKO IntraStain kit (DakoCytomation, Glostrup, Denmark) according to the manufacturer's instructions. Cells were then stained with conformation-specific monoclonal antibody against BAK (y164; Abcam, Cambridge, MA, USA) or isotype-matched control antibody for 30 min at room temperature, followed by incubation with Alexafluor 488-labeled chicken anti-rabbit secondary antibody (Molecular Probes, Eugene, OR, USA) for 30 min on ice in the dark. After the washing step, conformational change of BAK was analyzed by a FACScan flow cytometer.

### Western blot analysis and immunoprecipitation

Cells were solubilised in lysis buffer (phosphate-buffered saline solution (PBS), 1 × cell lysis buffer (Cell Signaling Technology, Danvers, MA, USA), 1 × protease inhibitor (Roche), and 1 × phosphatase inhibitor cocktails I and II (Calbiochem, San Diego, CA, USA)), and incubated for 30 min on ice. Total protein (20 *μ*g) was separated by sodium dodecyl sulphate polyacrylamide gel electrophoresis (SDS–PAGE), immunoblotted with appropriate antibodies, and reacted with enhanced chemiluminescence reagent (Amersham Biosciences, Piscataway, NJ, USA); signals were detected by a luminescent image analyser (LAS-1000 plus; Fujifilm, Tokyo, Japan). The anti-*α*-tubulin or anti-*β*-actin blot was used in parallel as a loading control. For immunoblotting, the following antibodies were used: p21^Cip1/WAF1^, p27^KIP1^, and Mcl-1 (BD-Pharmingen, San Diego, CA, USA); p53 (DO-7; Dako, Carpinteria, CA, USA); Noxa (Calbiochem); *α*-tubulin (Sigma-Aldrich, St Louis, MO, USA); Puma (Upstate Biotechnology, Lake Placid, NY, USA); LC-3 (MBL, Nagoya, Japan); ubiquitin (Santa Cruz Biotechnology, Santa Cruz, CA, USA); and Hsp70, c-Raf, Akt, ERK1/2, phosphorylated-ERK1/2 ^Thr202/Tyr204^(p-ERK1/2), cyclin D1, phosphorylated Rb^Ser780^ (p-Rb), Bim, BAK, cleaved caspase-9, cleaved caspase-3, *β*-actin, and horseradish peroxidase-linked anti-mouse and anti-rabbit IgG (all from Cell Signaling Technology). Protein lysates were subjected to immunoprecipitation using anti-Mcl-1 (Santa Cruz Biotechnology).

### Hsp90 binding assay

The Hsp90*α* inhibitor screening assay kit with Hsp90*α* recombinant enzyme and fluorescein isothiocyanate (FITC)-labelled geldanamycin was used (BPS Bioscience, San Diego, CA, USA). The competition of fluorescence-labelled geldanamycin for binding to purified recombinant Hsp90*α* was measured by Flex Station 3 (Molecular Devices, Sunnyvale, CA, USA).

### Morphological observation

U2OS-H2BK-EGFP cells (2.0 × 10^5^ per ml) were cultured in a 35-mm dish and treated with 5 *μ*M GUT-70 or DMSO only. Each dish was placed on the stage of a light microscope equipped with a digital camera (BZ-8000; Keyence, Osaka, Japan) at 37 °C under a humidified atmosphere of 5% CO_2_. Video images were collected over the period from 12 to 48 h after treatment.

### mRNA quantification by real-time reverse-transcriptase PCR (RT–PCR)

Total RNAs were extracted from cells with the RNeasy Mini Kit (Qiagen, Hilden, Germany). First-strand cDNA synthesis was performed with oligo(dT) as primer (Superscript II System; Invitrogen, Carlsbad, CA, USA). Real-time reverse-transcriptase PCR was performed by the Model 7500 Real-time PCR System (Applied Biosystems, Foster City, CA, USA). Expression of *Noxa* and *GAPDH* mRNA was detected by TaqMan Gene Expression Assays (*Noxa*: Hs00560402_m1, *GAPDH*: Hs99999905_m1; Applied Biosystems). The PCR cycle number that generated the first fluorescence signal above a threshold value (the threshold cycle; *C*_t_) was determined. The abundance of each transcript of *Noxa* relative to that of *GAPDH* was calculated as follows: relative expression=100 × 2 exp [−Δ*C*_t_], where Δ*C*_t_ is the mean *C*_t_ of the transcript of interest minus the mean *C*_t_ of the transcript for *GAPDH*. The *C*_t_ data from duplicate PCRs were averaged for calculation of relative expression.

### Statistical analysis

Cytotoxicity was assessed by the Chou-Talalay method (Chou and Talalay, 1984) using Calcusyn software (Biosoft, Cambridge, UK). The combination index (CI) values indicate degree of synergism: strong synergism (0.3–0.7), moderate synergism (0.7–0.85), and slight synergism (0.85–0.9).

## Results

### GUT-70 induces apoptosis and cell cycle arrest in MCL cells

**Figure 1:**
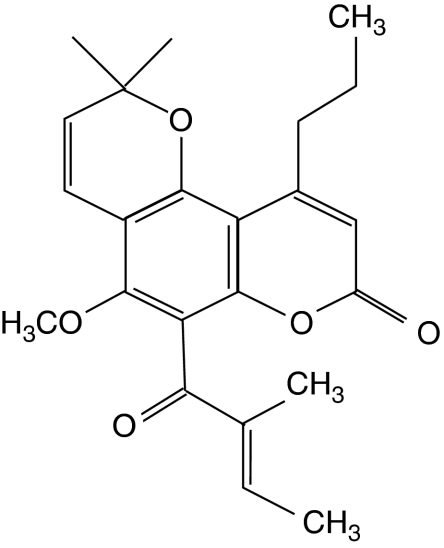
Chemical structure of GUT-70.

Treatment with GUT-70 (Figure 1) for 48 h resulted in dose-dependent cell growth inhibition detected by MTS cell proliferation assay (IC_50_: Granta 519, 6. 3 *μ*M; JVM-2, 4.5 *μ*M; Jeko-1, 1.7 *μ*M; MINO, 1.5 *μ*M).

**Figure 2:**
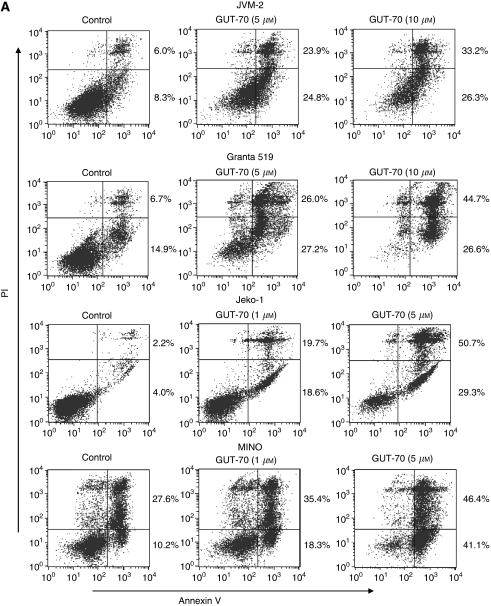

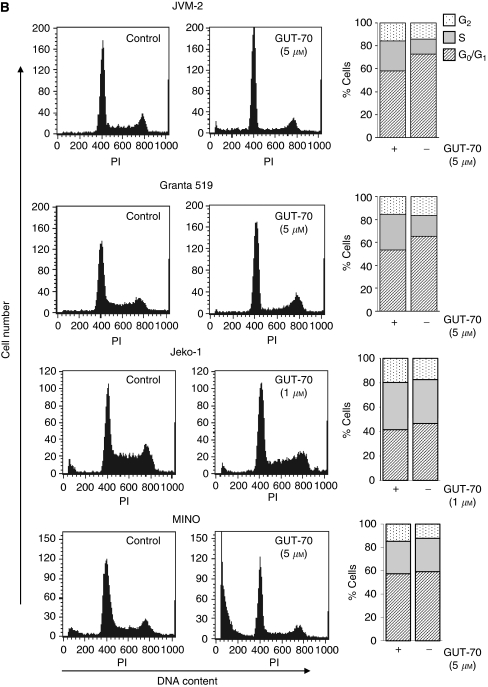
GUT-70-induced cell growth inhibition, apoptosis, and cell cycle arrest in MCL. (**A**) JVM-2, Granta 519, Jeko-1, and MINO cells were treated with the indicated concentrations of GUT-70 for 48 h, and the percentages of apoptotic cells were quantified by annexin V/PI staining. (**B**) Representative flow cytometric histograms of PI-treated cells after 24 h of GUT-70 treatment at indicated concentrations. The percentages of G_0_/G_1_-, S- and G_2_/M-phase cells were assessed in total viable cells (bar graphs).

To determine whether the inhibition of cell growth by GUT-70 was associated with apoptosis and/or cell cycle arrest, we conducted flow cytometric analysis of annexin V/PI-stained and PI-stained nuclei. As shown in Figure 2A, 48 h of GUT-70 treatment induced dose-dependent increases of annexin V positivity in all cell lines; this effect was more pronounced in mt-*p53-*bearing Jeko-1 and MINO cells than in wt-*p53-*bearing Granta 519 and JVM-2 cells (specific apoptosis by 5 *μ*M GUT-70: 40.3% for Granta 519, 40.1% for JVM-2, 78.8% for Jeko-1, 79.9% for MINO). The PI cell cycle histograms further demonstrated that GUT-70 increased the sub-G_1_ fraction in a time-dependent manner at a lower dose for mt-*p53* cells than for wt-*p53* cells; sub-G_1_ fractions at 24 and 48 h were 4.6 and 10.7% for Granta 519 (5 *μ*M GUT-70), 14.8 and 34.7% for JVM-2 (5 *μ*M), 5.2 and 19.3% for Jeko-1 (1 *μ*M), and 12.0 and 34.9% for MINO (1 *μ*M). Whereas GUT-70 impeded G_1_-S cell cycle progression in JVM-2 and Granta 519 cells, G_1_-S arrest was minimal in MINO and Jeko-1 cells (Figure 2B). These data suggest that GUT-70-induced cell growth inhibition resulted in part from cell cycle arrest at the G_0_/G_1_ checkpoint and in part from apoptosis induction.

### GUT-70 downregulates mutated p53 and cyclin D1 and accumulates p27

**Figure 3:**
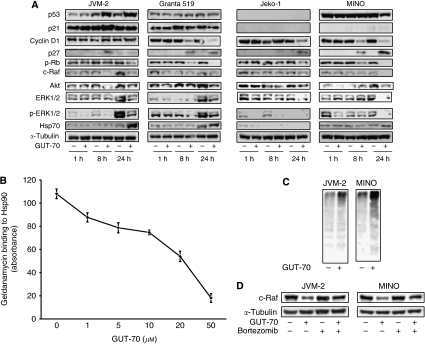
(**A**) GUT-70 effects on Hsp90 client proteins. MCL cells were treated with GUT-70 (JVM-2, 5 *μ*M; Granta 519, 5 *μ*M; Jeko-1, 1 *μ*M; MINO, 5 *μ*M) for indicated times. Cells were subjected to lysis and analysed by western blot. Western blot images are representative results from three independent experiments. (**B**) GUT-70 competitively inhibited geldanamycin binding to the Hsp90*α* subunit. The competition of GUT-70 with FITC-labelled geldanamycin for binding to purified recombinant Hsp90*α* was measured. Fluorescence was measured at *λ*ex 485 nm and at *λ*em 525 nm. Results shown are means±s.d. from three independent experiments. (**C**) GUT-70 induced protein ubiquitination. JVM-2 and MINO cells were treated with 5 *μ*M GUT-70 for 24 h, subjected to lysis, and immunoblotted for ubiquitin. Representative results are shown from three independent experiments. (**D**) Proteasome inhibitor bortezomib prevented GUT-70-mediated decreased expression of c-Raf. JVM-2 and MINO cells were treated with 5 *μ*M GUT-70 and/or 10 nM bortezomib for 24 h, subjected to lysis, and immunoblotted for c-Raf. Western blot images are representative results from three independent experiments.

We next investigated changes in cell cycle regulatory proteins associated with GUT-70 treatment. As shown in Figure 3A, GUT-70 induced p53/p21 accumulation in JVM-2 cells, but did not increase p53/p21 expression in Granta 519 cells. In Jeko-1 cells, basal p53/p21 expression was not detectable and was unaffected by GUT-70. Notably, expression of the overexpressed mt-p53 protein was reduced in MINO cells by 24 h exposure to GUT-70, without detectable p21 expression. The expression level of p27 was upregulated by GUT-70, irrespective of *p53* status. GUT-70 diminished the highly expressed cyclin D1 in all tested MCL cells except JVM-2, and resulted in substantial decreases in Rb phosphorylation in all tested cells (Figure 3).

### GUT-70 induces degradation of Hsp90 substrate proteins

The coumarin antibiotics have been reported to bind to Hsp90 (Marcu *et al*, 2000). To investigate whether GUT-70 has binding affinity for Hsp90, a competitive binding assay was performed using geldanamycin, a well-characterised ATP competitive inhibitor (Gooljarsingh *et al*, 2006). GUT-70 demonstrated dose-dependent inhibition of geldanamycin binding to Hsp90, which indicated the binding activity of GUT-70 to Hsp90 (Figure 3B). The degradation by GUT-70 of Hsp90 client proteins, such as Raf-1 and its downstream ERK1/2 and phospho ERK1/2, as well as Akt (Pratt and Toft, 2003; Zhang *et al*, 2004, 2005), was detected by western blot analysis in all tested MCL cells (Figure 3A). Cyclin D1 and mt-p53, the expression of which was repressed by GUT-70, are known substrate proteins of Hsp90 (Zhang *et al*, 2004; Muller *et al*, 2008). Furthermore, GUT-70 increased expression of Hsp70, a marker of Hsp90 inhibition (Elo *et al*, 2005; Bao *et al*, 2009), in Granta 519, JVM-2, and MINO cells. In Jeko-1 cells, however, Hsp70 was detected at a level insufficient to be reliable as a marker without further induction by GUT-70 (Figure 3A).

As the client proteins of Hsp90 chaperone molecule become misfolded and ubiquitinated by Hsp90 inhibition, and are then downregulated by proteasomal degradation (Issacs *et al*, 2003; Zhang *et al*, 2004), we next tried to determine whether GUT-70 induces protein ubiquitination followed by proteasomal degradation in wt*-p53-*expressing JVM-2 and mt*-p53-*expressing MINO cells. As expected, GUT-70 treatment elevated the level of protein ubiquitination (Figure 3C); subsequent treatment with proteasome inhibitor bortezomib prevented degradation of c-Raf by GUT-70 (Figure 3D). Taken together, these data indicate the interaction of GUT-70 with Hsp90 and the destabilisation of Hsp90 client proteins by GUT-70.

### Effect of GUT-70 on apoptosis-related proteins

**Figure 4:**
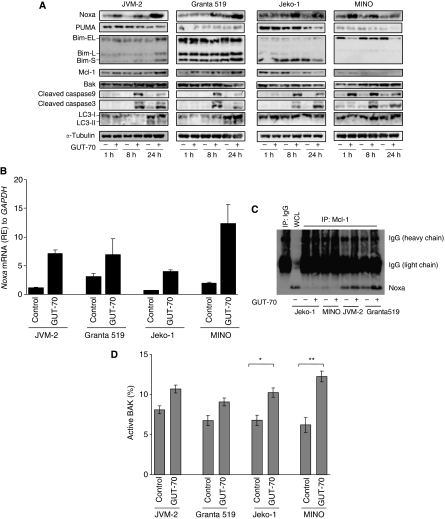
Modulation of apoptosis-related protein levels by GUT-70. MCL cells were treated with GUT-70 (JVM-2, 5 *μ*M; Granta 519, 5 *μ*M; Jeko-1, 1 *μ*M; MINO, 5 *μ*M) for the indicated times. (**A**) Cells were subjected to lysis, then apoptosis-related proteins and macroautophagy marker LC3 were analysed by western blot. Western blot images are representative results from three independent experiments. (**B**) Cells were harvested after 18 h treatment with GUT-70, and *Noxa* mRNA expression levels were detected by TaqMan RT–PCR analysis. The abundance of transcripts of *Noxa* relative to *GAPDH* transcripts was determined as described in Materials and Methods. Graphs show the representative data from two independent experiments with similar results. (**C**) Cells were treated with GUT-70 for 24 h, and Mcl-1 immunoprecipitation was performed as described in Materials and Methods. Total extracts were analysed by western blotting for Noxa. Western blot images are representative results from three independent experiments. (**D**) Cells were treated with GUT-70 for 24 h, then conformational changes in BAK were measured by intracellular flow cytometry as described in Materials and methods. To block the caspase activation-mediated conformational changes of BAK, cells were preincubated for 1 h with 100 *μ*M Z-VAD-FMK. Data represent duplicate experiments. ^*^P<0.01; ^**^P<0.05. RE, relative expression.

To characterise the mechanism of GUT-70-induced cell death in MCL cells, we analysed the expression of apoptosis-related Bcl-2 family proteins, the BH3-only proteins Noxa, Puma, and Bim, and the other Bcl-2 family proteins, Mcl-1 and BAK, in MCL cell lines. Results show that GUT-70 induced substantial accumulation of Noxa but not of Puma (Figure 4A). Mutant-*p53-*bearing MCL cells demonstrated earlier Noxa induction than wt-*p53* cells; peak induction of Noxa was observed after 1 h of GUT-70 treatment in MINO, after 8 h in Jeko-1, and after 24 h in JVM-2 and Granta 519 cells. GUT-70 induced upregulation of *Noxa* mRNA levels in all tested cells (Figure 4B).

After 24 h of GUT-70 treatment, levels of antiapoptotic protein Mcl-1 were increased in JVM-2 and Granta 519 cells, but decreased in Jeko-1 and MINO cells (Figure 4A). It is known that Noxa binds preferentially to Mcl-1 (Warr and Shore, 2008), triggers BAK or Bim release from Mcl-1, and then starts the mitochondrial apoptotic pathway (Willis *et al*, 2005; Hauck *et al*, 2009). Concordantly, we detected coimmunoprecipitation between Noxa and Mcl-1 (Figure 4C) in JVM-2 and Granta 519 cells, both of which showed accumulation of Mcl-1 induced by GUT-70. Although total BAK expression levels remained, consistently independent of GUT-70 treatment (Figure 4A), flow cytometric analysis revealed a pronounced increase of activated BAK in MINO, moderate activation in Jeko-1, and only slight activation in JVM-2 and Granta 519 cells after GUT-70 treatment (Figure 4D), activities that are in inverse relation to Mcl-1 expression levels.

Proapoptotic BH-3-only protein Bim was induced by GUT-70 at 24 h in JVM-2 cells but not in the other cell lines (Figure 4A).

### GUT-70 does not induce macroautophagy

Increasing evidence indicates that autophagy is one of the important mechanisms of anticancer reagent-induced cell death (Tsujimoto and Shimizu, 2005). In mammals, three modes of autophagy have been identified: macroautophagy, microautophagy, and chaperone-mediated autophagy (Levine and Klionsky, 2004). To investigate the possibility that GUT-70 promotes macroautophagy, we examined the conversion of light chain 3 (LC3) from LC3-I to LC3-II, a marker of autophagosome formation (Kabeya *et al*, 2000). Whereas LC3-II was moderately induced by a low serum culture condition (5%, 40 h) in wt-*p53-*expressing JVM-2 and Granta 519 cells, there was no change in accumulation of LC3-II following further treatment with GUT-70 (24 h). In mt-*p53-*bearing MINO and Jeko-1 cells, neither serum starvation nor GUT-70 treatment induced LC3-II accumulation (Figure 4A).

To assess the morphological changes induced by GUT-70, U2OS-H2BK-EGFP cells were sequentially photographed after exposure to GUT-70. Cells underwent morphological alterations, including cytoplasmic swelling and vacuolisation after 24 h of GUT-70 exposure (cellular oncosis), and cell death peaked at 36 h (secondary necrosis) (Supplementary Material 1 for Quick-Time movies) (Majno and Joris, 1995; Lemasters *et al*, 1998; Van Cruchten and Van Den Broeck, 2002).

### Combination of GUT-70 with bortezomib or doxorubicin has synergistic effects on MCL growth inhibition

**Figure 5:**
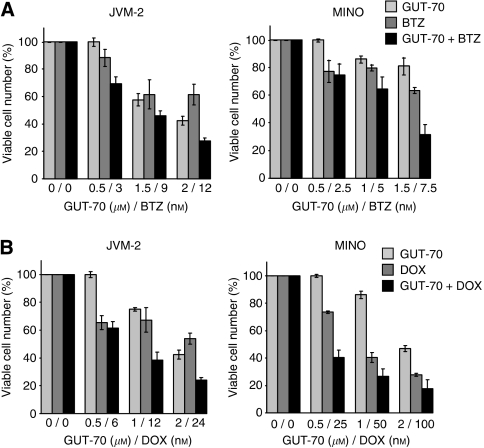
Synergistic interaction between GUT-70 and bortezomib or doxorubicin in MCL cells. JVM2 and MINO cells were cultured in the presence of escalating doses of GUT-70 and bortezomib (BTZ) (**A**) or GUT-70 and doxorubicin (DOX) (**B**) at a fixed ratio. After 48 h, viable and dead cells were identified using the Trypan blue dye exclusion method. Results are expressed as means±s.d. percentage of viable cell numbers in control cells.

To determine whether GUT-70 potentiates the commonly used chemotherapeutic agents, we assessed the effects of combinations of GUT-70 with bortezomib, a selective inhibitor of the 26S proteasome, or doxorubicin, a conventional chemodrug for MCL, on viability of wt*-p53* JVM-2 and mt-*p53* MINO cells. As shown in Figure 5A, both of these combination treatments had observable synergistic effects in both cell types 48 h after exposure. The averaged CI values of GUT-70/bortezomib treatment were 0.59 for JVM2 and 0.73 for MINO; for GUT-70/doxorubicin, 0.37 for JVM2 and 0.35 for MINO, indicating strong and moderate synergism, respectively.

## Discussion

The natural product-derived tricyclic coumarin GUT-70 exhibited single-agent antiproliferative and proapoptotic activities against MCL cell lines as a novel Hsp90 inhibitor. GUT-70's dose-dependent inhibition of geldanamycin binding to Hsp90*α* indicates that GUT-70 has direct binding activity to Hsp90, by which GUT-70 induces conformational change in the Hsp90 molecule and interferes with its binding of geldanamycin. This finding agrees with that of previous studies showing that coumarin antibotic novobiocin binds to the Hsp90 C-terminal ATP binding site and affects the binding of geldanamycin at the Hsp90 N-terminal domain through close interaction between amino and carboxy termini in solution (Csermely *et al*, 1998; Hartson *et al*, 1999; Marcu *et al*, 2000; Donnelly *et al*, 2008). GUT-70 induced depletion of Hsp90 client proteins mt-p53, Raf-1, cyclin D1, and Akt, and increased Hsp70, a marker of Hsp90 inhibition; these findings, along with the ubiquitin-dependent proteasomal degradation of Hsp90 client proteins, suggest that GUT-70 functions as an Hsp90 inhibitor.

It is important that mt-*p53-*expressing MCL cells were more sensitive to GUT-70-induced apoptosis than wt-*p53-*bearing MCL cells. In mt-*p53* cells, prominent GUT-70-induced apoptosis was accompanied by minimal cell cycle arrest, which is consistent with a previous report of G_2_/M checkpoint abrogation in p53/p21-impaired cells through downregulation of Chk1 and Wee1 by Hsp90 inhibitor that resulted in premature mitotic entry and mitotic death (Tse *et al*, 2009).

Furthermore, GUT-70 induced the most pronounced apoptosis in MINO cells in which GUT-70 treatment depleted overexpressed mt-p53. mt-p53 is known to confer the additional ‘gain of function’ as the transcription regulator. Transcriptional activation by mt-p53 has been reported for *MDR1* (Sampath *et al*, 2006), c-*MYC* (Frazier *et al*, 1998), or *GRO1* (Yan and Chen, 2009), resulting in cell proliferation, antiapoptosis, and tumourigenicity (Blandino *et al*, 1999). GUT-70-induced degradation of mt-p53 may successfully repress these oncogenic transcriptional activations.

Another important finding of this study is the prominent *p53*-independent Noxa upregulation by GUT-70. Whereas Noxa had been proposed to be a critical mediator of *p53*-dependent apoptosis (Oda *et al*, 2000), *p53*-independent upregulation of Noxa has been described in MCL and B-cell chronic lymphocytic leukaemia (Pérez-Galán *et al*, 2006; Smit *et al*, 2007). Furthermore, GUT-70 induced Noxa protein accumulation extremely early (1 h) in mt-*p53-*bearing MINO cells, indicating independence from transcriptional gene induction. Recently, Noxa degradation by direct interaction with a spliced isoform of the Kruppel-like tumour suppressor (KLF6-SV1) (Difeo *et al*, 2009), or by posttranscriptional stabilisation/destabilisation of *Bim* mRNA (Matsui *et al*, 2007), has been reported. Our findings indicate the possibility of posttranscriptional Noxa stabilisation by GUT-70, which requires further elucidation.

The preferred binding partner of Noxa is the multidomain antiapoptotic Bcl-2 family member Mcl-1. In response to apoptotic stimuli, Noxa binds to Mcl-1, which ultimately leads to activation of BAK by releasing BAK from the BAK−Mcl-1 complex, and triggers BAK-mediated cell death (Chen *et al*, 2005; Warr and Shore, 2008) (Figure S, Supplementary Material 2 (Kuroda and Kimura, 2007)). The balance between Noxa and Mcl-1 is proposed to determine cell fate as death versus survival (Mei *et al*, 2007). GUT-70-induced BAK activation and sequential apoptosis were associated with Mcl-1 accumulation levels; high levels in less-sensitive wt-*p53* cells and low levels in highly sensitive mt-*p53* cells were consistent with previous reports (Pérez-Galán *et al*, 2006; Mei *et al*, 2007).

Autophagy is known to promote both autophagic cell death and cell survival (Kamitsuji *et al*, 2008). Although GUT-70 did not affect autophagosome formation, Hsp90 clients have been shown to be degraded through chaperone-mediated autophagy (Shen *et al*, 2009). The role of GUT-70 in induction of chaperone-mediated autophagy requires further elucidation.

The observed morphological changes in GUT-70-treated cells (e.g., swelling cytoplasm) indicate cellular oncosis (Van Cruchten and Van Den Broeck, 2002), which shares certain mechanisms and alterations with apoptosis, such as loss of mitochondrial permeability and membrane potential (Lemasters *et al*, 1998).

Furthermore, our results demonstrate that GUT-70 can synergise the cytotoxic effects of the proteasome inhibitor bortezomib and the widely used genotoxic chemotherapeutic agent doxorubicin in MCL cells (Brody and Advani, 2006; Goy *et al*, 2009), regardless of p53 status. Previously, a combination of Hsp90 inhibitor geldanamycin and bortezomib was demonstrated to simultaneously disrupt Hsp90 and proteasome function, promote accumulation of ubiquitinated proteins, and enhance antitumour activity in human breast cancer cells (Mimnaugh *et al*, 2004, 2006). Whereas bortezomib induces longer-term remission (Goy *et al*, 2009), patients ultimately succumb to the poor clinical outcome, and there is a critical need to develop the most effective combination. The synergistic effects of GUT-70 and bortezomib may offer more efficacy and flexibility to the treatment of MCL with bortezomib. The antiproliferative effect of the combination of doxorubicin and GUT-70 was consistent with the previous findings for doxorubicin and Hsp-90 inhibitor 17-(dimethylaminoethylamino)-17-demethoxygeldamanycin (DMAG), which induced premature mitosis, followed by apoptosis, by bypassing the G_2_/M checkpoint in lymphoma cells (Robles *et al*, 2006). The synergy with doxorubicin suggests that addition of GUT-70 may allow reduction in the therapeutic dose of doxorubicin, which could potentially reduce its genotoxic side effects (Brody and Advani, 2006). A development of *in vivo* studies of these combination treatments for MCL is further required.

In conclusion, our results demonstrate that the novel anticancer agent GUT-70, a tricyclic coumarin, inhibits cell proliferation by depleting Hsp90 substrates cyclin D1, Akt, and Raf-1, and induces mitochondrial apoptotic cell death with upregulation of Noxa in MCL cells. Notably, these effects are substantially pronounced in MCL cells with mt-*p53*, a known negative prognostic factor for MCL. These findings suggest that GUT-70 has potential utility for the treatment of MCL.
