# Supplementary figures and images for: Antiproliferative and proapoptotic activity of GUT-70 mediated through potent inhibition of Hsp90 in mantle cell lymphoma
Source: Br J Cancer. 2010 Dec 7;104(1):91–100. doi: 10.1038/sj.bjc.6606007 (PMC3039813; doi:10.1038/sj.bjc.6606007)

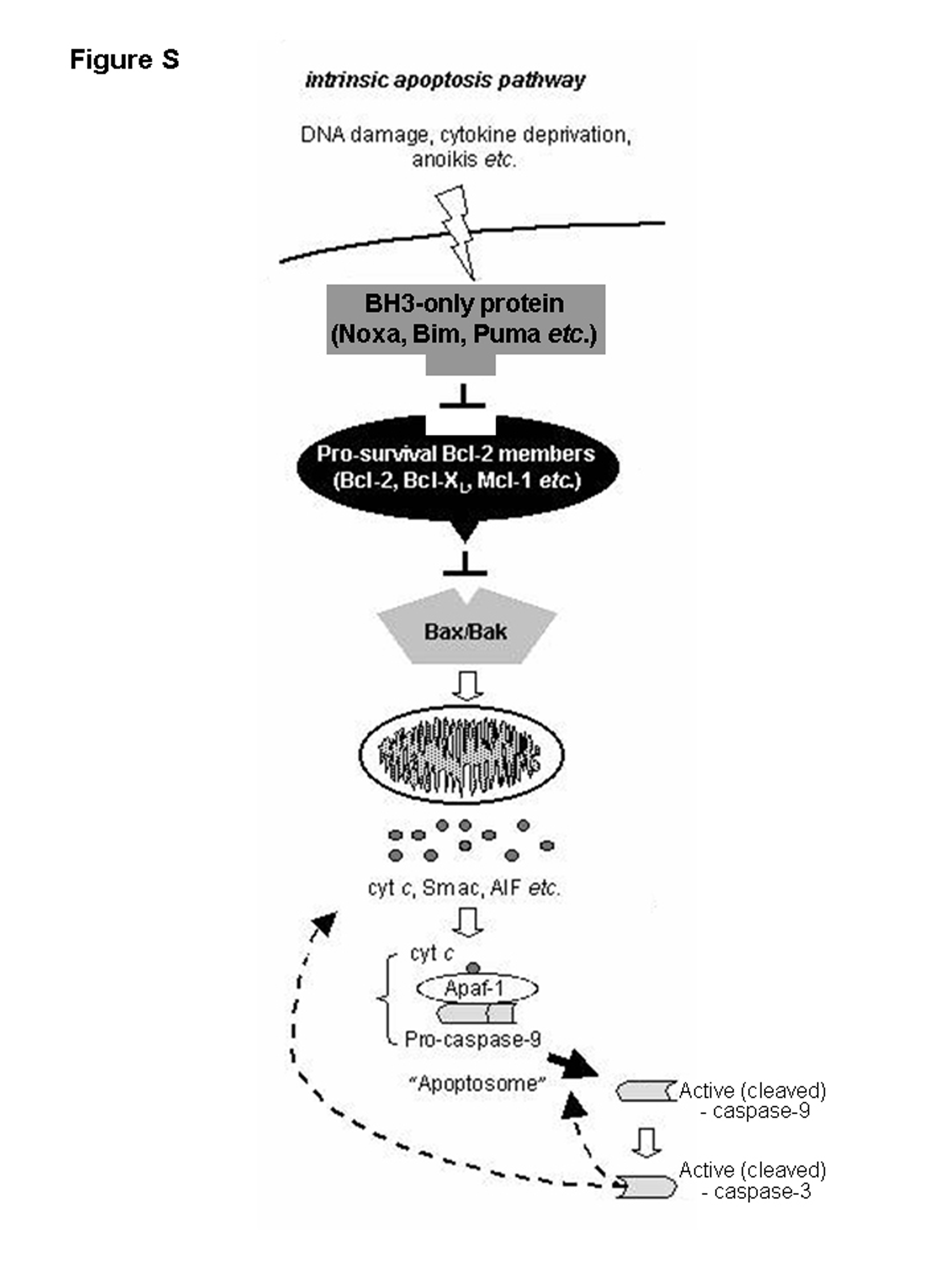

Supplement: Supplementary Figure 1 [file 6606007x1.tif]
