# Supplementary material for: Antiproliferative and proapoptotic activity of GUT-70 mediated through potent inhibition of Hsp90 in mantle cell lymphoma
Source: Br J Cancer. 2010 Dec 7;104(1):91–100. doi: 10.1038/sj.bjc.6606007 (PMC3039813; doi:10.1038/sj.bjc.6606007)
Supplement: Supplementary Material [file 6606007x4.doc]

**Supplemental Material 1**

**Video 1, 2. GUT-70–induced morphologic changes.** U2OS-H2BK-EGFP cells were cultured with or without 5 μM GUT-70. Real-time analysis by a light microscope equipped with a digital camera (BZ-8000) showed morphologic changes after 24 hours of GUT-70 exposure: swelling of the cytoplasm without visible nuclear breakdown. Video 1: control cells; Video 2: GUT-70-treated cells.

**Supplemental Material 2**

**Figure S. Apoptosis-initiating molecular cascades of the intrinsic apoptosis pathway.** Intrinsic apoptosis is initiated by activation of BH3-only proteins, which in turn binds and inactivates prosurvival Bcl-2 family proteins, then activates Bax/Bak on mitochondria. As a consequence, mitochondrial proteins, such as cytochrome *c*, are released, and caspase-9 and then effector caspase-3 are activated.
